# Description of a Rare Case of Nodular Fasciitis of the Apical Aspect of the Upper Buccal Sulcus

**DOI:** 10.1155/2016/4231683

**Published:** 2016-03-15

**Authors:** Ana Amélia Souza, Eldon Guttenberg Cariri Neto, Vera Cavalcanti de Araújo, Fabricio Passador-Santos, Maria Teresa de Seixas Alves, Andresa Borges Soares

**Affiliations:** ^1^Department of Oral Pathology, Sao Leopoldo Mandic Institute and Research Center, Rua José Rocha Junqueira13, Ponte Preta, 13045-755 Campinas, SP, Brazil; ^2^Department of Pathology, Escola Paulista de Medicina, Rua Botucatu 740, Vila Clementino, 04023-900 Sao Paulo, SP, Brazil

## Abstract

This report describes a rare case of nodular fasciitis (NF) of the oral cavity, discussing the clinical, histological, and immunohistochemical characteristics. Histopathologic diagnosis of this type of lesion can be challenging due to its differential diagnosis, which principally includes sarcoma. The patient presented with a painless, well-defined nodule, reported as increasing in size, located at the apical aspect of the upper left buccal sulcus. Histologically, the lesion revealed spindle cell proliferation arranged in fascicles, while immunohistochemistry demonstrated positivity for smooth muscle actin. Eight months after complete surgical excision, no signs of local recurrence have been observed.

## 1. Introduction

Nodular fasciitis (NF) is a benign reactive soft tissue lesion, frequently found in the subcutaneous tissues and muscle fascia, which shows proliferation of myofibroblasts and fibroblasts. While NF has been frequently reported at many anatomical sites, namely, the upper extremities, the trunk, head and neck, and, lastly, the lower extremities, it is extremely rare in the oral cavity [1, 2].

NF was first described by Konwaler et al. as a subcutaneous pseudosarcomatous fibromatosis and has subsequently been nominated as infiltrative fasciitis and pseudosarcomatous fasciitis [3–5]. Several authors believe that the lesion represents a reactive or inflammatory condition triggered by local injury or infection [6]. However, the presence of a reciprocal translocation involving chromosome 15 has been recently described, which may categorize this lesion as a true neoplasm [7–9]. Erickson-Johnson et al. [10] confirmed the presence of the translocation in 44 cases of NF. The authors also identified a new translocation-induced fusion gene,* MYH9-USP6*, and postulated that NF could be the first example of a self-limiting human lesion that can be characterized by a recurrent somatic gene fusion event.

The histopathological diagnosis of NF can be challenging, as its differential diagnosis includes other significant soft tissue lesions, such as sarcoma. NF is characterized by myofibroblast and fibroblast proliferation. Immunohistochemical panels are often needed to aid in diagnosis, with NF showing positivity for vimentin, smooth muscle actin, and muscle specific actin [11–15].

The present report describes the clinical and histopathologic features of a rare case of nodular fasciitis of the oral cavity.

## 2. Case Report

A 17-year-old white female presented to a private dental clinic complaining of a painless swelling to the apical aspect of the upper left buccal sulcus, which had increased in size over the preceding seven weeks. The medical history was otherwise unremarkable.

**Figure 1:**
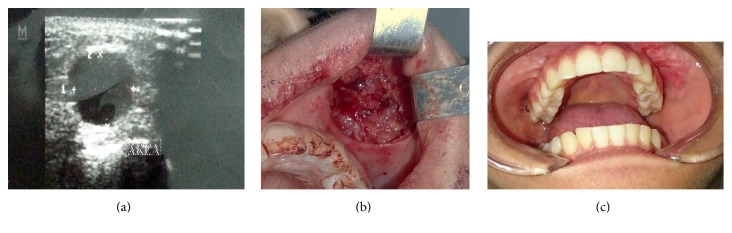
(a) Ultrasound examination showing the presence of a hypoechoic tumoral mass. (b) Transoperative view. (c) Follow-up at 8 months.

Initial inspection revealed a gross asymmetry of the face, with no palpable lymph nodes. Intraoral examination demonstrated a well-defined, rubbery, painless, nonulcerated mass at the apical aspect of the upper left buccal sulcus. Ultrasound examination showed a rounded hypoechoic mass (Figure 1(a)). An excisional biopsy of the soft tissue mass was performed, and a clinical diagnosis of a benign tumor was suggested. The lesion was completely excised during biopsy (Figure 1(b)). The surgical specimen consisted of an irregular, fibrous, yellow soft tissue mass.

**Figure 2:**
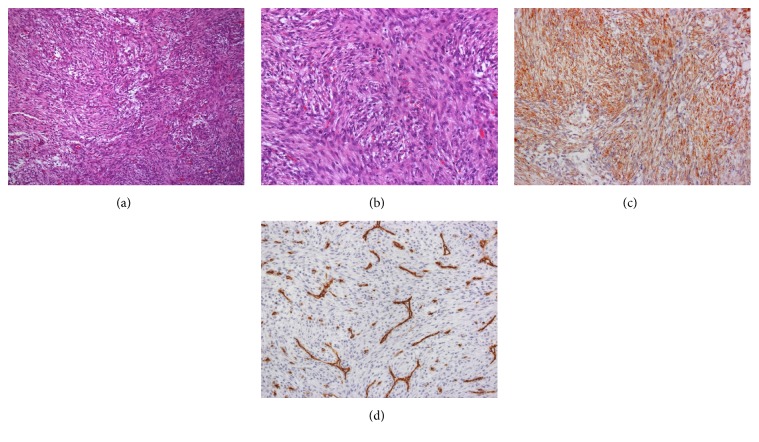
(a) Proliferation of spindle cells arranged in fascicles (Hematoxylin and Eosin stain, original magnification 10x). (b) Cells of nodular fasciitis with elongated shape, oval nuclei, and eosinophilic cytoplasm (Hematoxylin and Eosin stain, original magnification 40x). (c) Immunohistochemical stain showing strong positivity for smooth muscle actin (smooth muscle actin stain, original magnification 20x). (d) Immunohistochemistry stain showing negativity for CD34 (CD34 stain, original magnification 20x).

The specimen was fixed in 10% buffered formalin, with paraffin sections being prepared for light microscopy following standard procedures. Microscopic examination of the Hematoxylin and Eosin (H&E) stained sections revealed spindle cell proliferation arranged in fascicles. In general, the cells were elongated, with oval nuclei and eosinophilic cytoplasm. Rare mitotic figures were observed, yet no atypical mitoses were present. Scarce discrete cell pleomorphism was observed, with myxoid areas also present. A chronic inflammatory process scattered throughout the lesion was observed (Figures 2(a) and 2(b)).

Immunohistochemistry revealed spindle cell positivity to the smooth muscle actin (clone 1A4) (Figure 2(c)) antibody, while an absence of reactivity was seen for CD34 (clone M 7165) (Figure 2(d)), AE1/AE3 (clone M3515), and S-100 (clone Z0311).

No sign of local recurrence has been observed after eight months of follow-up (Figure 1(c)).

## 3. Discussion

The case reported, herein, fulfills the clinical, histological, and immunohistochemical criteria for the diagnosis of NF, a rare benign tumor of the oral cavity. The challenge in diagnosis of oral NF comes from the fact that the lesion is rare in the oral cavity, as well as the fact that it has a significant histopathologic differential diagnosis [11].

According to the literature, NF is typically diagnosed in young adults between the ages of 20 and 40 years [12–15]. Lima et al. [16] published a literature review of oral cavity NF over the past 10 years, which revealed an average age of 34 years, with a maximum age of 50 and minimum of 8 years. In the present case, the lesion arose in a 17-year-old female, which is considered younger age than that previously described for patients with NF.

The clinical features of NF are rather nonspecific, with it usually being described as a well circumscribed nonencapsulated mass, soft and elastic in consistency, which firmly adheres to the underlying tissues [13, 14]. Oral NF lesions are most common in the buccal and labial mucosa, as well as tongue, with the parotid gland and floor of the mouth also being affected [2, 12–18]. NF can present as multiple nodules in a single area, with multiple anatomic sites rarely affected concurrently [13]. In the present case, the lesion was a well-defined, rubbery, painless, nonulcerated mass located at the apical aspect of the upper left buccal sulcus. Ultrasound of the lesion revealed a round, hypoechoic mass.

NF is characterized by rapid growth, which often leads to tissue distortion, mimicking its malignant counterpart [11, 12]. In the present case, the lesion was reported as having increased in size over the course of seven weeks, which corroborates the literature [11–14, 16, 18, 19].

Histologically, NF is classified into three subtypes, according to the tissue plane involved: subcutaneous, intramuscular, and fascial. The lesion is usually attached to the fascia from which it arises and extends into the subcutaneous fat in an irregular fashion. Occasionally, it may also arise from the fibrous septa of the subcutaneous fat, only secondarily extending to the fascia [12, 16]. NF is usually a well circumscribed yet nonencapsulated mass composed of fibroblastic and myofibroblastic proliferation arranged in short fascicles and untidy bundles. Typical histologic findings include a myxoid and, in some cases, highly cellular stroma, exhibiting abundant mitotic activity, with the absence of cellular atypia. Myxoid degeneration may also be observed, while other areas are more fibrotic and hyalinized, displaying evidence of adjacent microhemorrhage with granulation tissue. Moreover, some lesions may display multinucleated giant cells [11–14]. The histological features of the present case are similar to those previously described. The lesion consisted of spindle cells arranged in fascicles, exhibiting oval nuclei and eosinophilic cytoplasm. While rare mitotic figures were observed, there were no atypical mitoses. Scarce discrete cellular pleomorphism was also observed. Myxoid areas were present, with a chronic inflammatory process and erythrocytes found scattered throughout the histological sections.

Despite the histopathological characteristics of the present case being similar to those of other NF cases, immunohistochemistry was an essential aid for the final diagnosis. In the literature, the immunohistochemical profile of NF includes positivity for vimentin, *α*-smooth muscle actin, and muscle specific actin, demonstrating the myofibroblastic differentiation of the spindle cells [11, 12, 16]. NF has also been described as being negative for CD34, cytokeratins, desmin, and the S-100 protein [5, 11, 12, 16]. In the present case, the spindle-like cells were positive for smooth muscle actin and negative for cytokeratin, CD34, and S100.

NF is a challenging diagnosis owing to its unusual clinical features, as well as its histological features, which are similar to other lesions [13, 14, 16]. Due to its rapid growth and the presence of mitoses and spindle cells and its rich cellularity, the differential diagnoses of sarcoma, including sarcomatoid carcinoma, fibrosarcoma, and leiomyosarcoma, are important [20]. It is important to highlight, however, that sarcoma is characterized by its prominent nuclear atypia, long, sweeping bundles, herringbone or storiform arrangement, atypical mitoses, and necrotic background, none of which are found in NF, as corroborated by the present report [21].

NF should also be distinguished from highly cellular lesions, such as fibrous histiocytoma, which displays dense fascicles of fibroblastic cells, chronic inflammation, lipophages, and giant cells. The myxoid appearance of NF can lead to a misdiagnosis of myxoma, myofibroma, fibrous histiocytoma, and fibromatosis or malignant peripheral nerve sheath tumor [22]. Myxoma, myofibroma, fibrous histiocytoma, and fibromatosis can, however, be distinguished from nodular fasciitis by their growth pattern and cytological features [13, 23].

While the treatment of choice for NF is surgical excision [12, 13, 16, 17], Yanagisawa and Okada [24] and de Carli et al. [20] have described total and partial spontaneous regression, respectively. Following surgical excision, recurrence is extremely rare, even when the excision is incomplete. In the current case report, surgical excision was performed and, after eight months of follow-up, no sign of recurrence has been observed.

In conclusion, NF is a benign lesion that can occur at any anatomical site, yet it is rare in the oral cavity. Despite its rarity, the dental surgeon and oral pathologist must have an adequate understanding of the lesion in order to establish an accurate diagnosis and offer appropriate treatment.
